# Effectiveness and safety of inspiratory muscle training in patients with pulmonary hypertension: A systematic review and meta-analysis

**DOI:** 10.3389/fcvm.2022.999422

**Published:** 2022-11-29

**Authors:** Zeruxin Luo, Hong Qian, Xiu Zhang, Yuqiang Wang, Jing Wang, Pengming Yu

**Affiliations:** ^1^Department of Rehabilitation Medicine Center, West China Hospital, Sichuan University, Chengdu, Sichuan, China; ^2^Department of Cardiovascular Surgery, West China Hospital, Sichuan University, Chengdu, Sichuan, China

**Keywords:** pulmonary hypertension, inspiratory muscle training, maximal inspiratory pressure, exercise capacity, quality of life

## Abstract

**Background:**

Inspiratory muscle training (IMT) is a simple and well-tolerated physical therapy that increases respiratory muscle strength and relieving the degree of dyspnea and fatigue. Therefore, it may be used as a transitional modality before exercise training or as a specific physical therapy intervention for those who are diagnosed with respiratory muscle weakness. However, the current evidence on IMT in pulmonary hypertension (PH) patients is inconclusive. The purpose of this systematic review and meta-analysis was to summarize the current role of IMT in this group of patients.

**Methods:**

PubMed, EMBASE, and Cochrane databases were searched through May 2022. Trials examining the feasibility and effectiveness of IMT in PH patients. Outcome measures included adverse events, training adherence and compliance, maximum inspiratory pressure (MIP), maximum expiratory pressure (MEP), forced vital capacity (FVC%), forced expiratory volume in 1 s (FEV_1_%), FEV_1_/FVC%, 6 min walk distance (6MWD), Peak VO_2_, dyspnea, and fatigue perception after the IMT training program. Only randomized controlled trials were included. The Cochrane Risk of Bias tool for controlled trials was adopted to assess study quality. Statistical heterogeneity was evaluated with the chi-square test and *I*^2^ statistic. Mean differences and 95% confidence intervals (CIs) were estimated.

**Results:**

We ultimately identified four studies that met the criteria. These studies comprised 80 patients with 16 males and 64 females. The mean age was 53.25. The main types of PH were group I (pulmonary arterial hypertension, 95%) and group IV (chronic thromboembolic PH, 5%). No severe adverse events were reported in the included studies. IMT had a significant effect on improving MIP (18.89 cmH_2_O; 95% CI: 9.43–28.35, *P* < 0.001) and MEP (8.06 cmH_2_O; 95% CI: 2.39–13.73; *P* = 0.005), increase in the 6MWD (30.16 m; 95% CI: 1.53–58.79; *P* = 0.04). No significant improvement was found in pulmonary function (*P* > 0.05), and uncertain effect on the quality of life (QoL) score.

**Conclusion:**

Based on currently limited evidence, IMT is an effective physical therapy for increasing respiratory muscle function and exercise capacity, but still a lack of evidence on dyspnea and fatigue levels, pulmonary function, and QoL in PH patients. There are reasons to believe that IMT is a promising intervention in PH patients, enriching rehabilitation options and serving as a bridge before formal exercise training. It is expected that IMT will play an important role in the future clinical pathway of physical therapy for this group of patients.

**Systematic review registration:**

[https://www.crd.york.ac.uk/PROSPERO/logout.php], identifier [CRD42022335972].

## Introduction

Pulmonary hypertension (PH) is an uncommon but devastating disorder characterized by obliterative pulmonary vascular remodeling, leading to a progressive increase in pulmonary vascular resistance and right heart failure (1). The prevalence of PH is about 1% and rising to 10% in the older population all over the world (2). Left heart or lung disease is the most common cause of PH. PH-induced decrease in pulmonary vasculature distensibility leads to a marked increase in mean pulmonary arterial pressure (mPAP) during exercise. This resulted in decreased pulmonary blood flow and cardiac output, which further impaired exercise capacity (3). This may further aggravate the symptoms of fatigue and dyspnea, impair physical function, and ultimately increase the risk of mortality (4).

An accumulation body of evidence demonstrates that exercise-based cardiac rehabilitation can relieve clinical symptoms and increase exercise capacity and quality of life (QoL) in PH patients (5). The latest European Society of Cardiology (ESC) and the European Respiratory Society(ERS) guidelines recommend that supervised exercise training should be considered in PAH patients with optimal drug treatment (Class I, evidence Level A) (6). However, the participation rate in clinical practice is not as high as desired (7). Increased dyspnea, feelings of fatigue, or inability to tolerate the intensity of exercise may be the reasons why PH patients are unable to participate in exercise training in clinical practice (8). Therefore, it is important to find a physical therapy technique for PH patients that can transition to regular exercise training and allows similar effects as exercise training.

Inspiratory muscle training (IMT) is a simple and well-tolerated physical therapy approach that may be a good choice. Previous studies have clarified the feasibility and effectiveness, especially on inspiratory muscle strength, functional capacity, and QoL in heart failure patients (9). In fact, respiratory muscle weakness, mainly the diaphragm, is an important factor in the reduced exercise capacity of patients with chronic respiratory and cardiovascular diseases (10, 11). The same situation exists in PH patients (12). Kabitz et al. (13) found maximum inspiratory pressure (MIP) and maximum expiratory pressure (MEP) were significantly lower in PAH patients and were associated with exercise intolerance. It is suggested that the potential role of respiratory muscle dysfunction may be a contributing factor to exercise intolerance in PAH patients. During exercise, patients with respiratory muscle weakness are prone to increased work of breathing, altered ventilatory response, and increasing dyspnea, which may ultimately lead to exercise intolerance (14). And as a form of muscle strength training, IMT can induce adaptive changes in respiratory muscle structure by increasing the strength or endurance of the diaphragm-based inspiratory muscles and other inspiratory support muscles, conferring higher strength, and fatigue resistance, thus enabling patients to maintain higher ventilation volumes while improving gas exchange (15, 16). This may be the underlying mechanism by which IMT improves the degree of dyspnea, exercise capacity, and QoL in PH patients.

To date, a few studies have focused on the safety, feasibility, and effectiveness of IMT in PH patients (17, 18). Saglam et al. (17) first reported that IMT can significantly improve inspiratory muscle strength, exercise capacity, dyspnea, and fatigue in PH patients (*P* < 0.05). However, there is no systematic review focused on the application of IMT in PH patients. Considering that, this systematic review and meta-analysis were to explore the safety and feasibility of isolated IMT in PH patients. It also further analyses the effects of this intervention on respiratory muscle function, pulmonary function, dyspnea, fatigue, exercise capacity, and QoL, intending to find a safe and effective transitional treatment option prior to exercise training for this group of patients.

## Methods

This systematic review and meta-analysis were conducted in accordance with the Preferred Reporting Items for Systematic Reviews and Meta-Analyses (PRISMA) guidelines and were registered in PROSPERO (CRD42022335972).

### Searching strategy

PubMed, EMBASE, and Cochrane databases were searched from the establishment of the database to May 2022. Search strategies consisted of two components: participants (PH, pulmonary arterial hypertension, pulmonary vascular disease, PH) and interventions (respiratory muscle training, inspiratory muscle training, expiratory muscle training, IMT, RMT), the detailed search method and PICOs format are shown in Supplementary Tables 1, 2. We also hand-searched the reference lists of candidate articles to find the articles that might have been missed during the literature search. All titles and abstracts were independently screened by two authors. For conflicting evaluations, the third senior author was consulted to solve the dispute and a final decision was made by most votes.

### Selection criteria

Studies were included according to the following inclusion criteria: (1) subjects aged >18 years with PH based on clinical diagnosis (19); (2) randomized control trial investigating the effects of isolated IMT, compared with sham IMT or no IMT(control group); (3) one of the following results must be reported: MIP, MEP, forced expiratory volume in 1 s (FEV_1_), forced vital capacity (FVC), FEV_1_/FVC%, 6 min walk distance (6MWD), QoL, dyspnea and fatigue perception, feasibility, and safety. Studies were excluded if they were: (1) meeting abstracts, reviews, non-human trials, protocols, observational studies, and case reports; (2) exercise training or other types of physical therapy were combined.

### Data extraction

Two authors independently extracted data with *a priori*-developed data extraction form. The following data were collected: (1) demographic and clinical information of study population such as age, gender, New York Heart Association Class (NYHA) or Word Health Organization Functional Class (WHO-FC), mPAP; (2) detailed intervention parameters including training type, frequency, duration, and intensity; (3) baseline and endpoint outcomes including MIP, MEP, 6MWD, Peak VO_2_, FVC, FEV_1_, Borg Score, QoL, and, adverse events.

### Quality assessment

Cochrane Risk of Bias tool for controlled trials was adopted to assess study quality. Aspects of random sequence generation, allocation concealment, blinding of participants and personnel, blinding of outcome assessments, incomplete outcome data, selective reporting, and other biases were evaluated. Each domain of these studies was assigned with either a low, high, or unclear risk of bias.

### Statistical analysis

Continuous data were presented with mean and standard deviation (SD). Data integration was conducted following the standardized procedures. Means at baseline were calculated by combining means of the intervention and control groups, weighted by the number of participants in each study group. SDs at baseline were estimated by combining SDs of the intervention and control groups with the following formula: SD = √[(N1-1) × SD12 + (N2-1) × SD22 + N1 × N2/N1 + N2 × (M12 + M22-2 × M1 × M2)] and weighted by the number of participants in each study group (20).

Review Manager 5.2 (The Nordic Cochrane Center, Copenhagen, Denmark) was used to conduct meta-analysis for included studies if at least two studies assessing a specific outcome with a similar instrument. Statistical heterogeneity was evaluated with the chi-square test and *I*^2^ statistic (21). The statistic was categorized as (1) *I*^2^ = 0–24%: low; (2) *I^2^* = 25–49%: moderate; (3) *I*^2^ = 50–74%: substantial; and (4) *I*^2^ = 75–100%: considerable heterogeneity. As we expected to generate substantial statistical heterogeneity, analyses were performed with a random or fixed effect model according to the specific heterogeneity. Variables such as MIP was analyzed for their positive mean difference with 95% confidence intervals (CIs) indicating the beneficial effects of IMT as compared to the control interventions.

## Results

### Study selection

**FIGURE 1:**
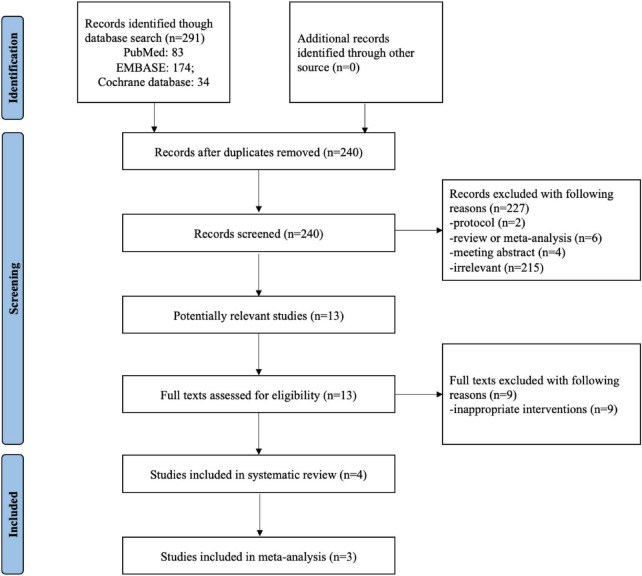
Flow chart of selecting studies for inclusion in systematic review and meta-analysis.

Figure 1 summarized the process of identifying eligible studies. 291 publication items were identified during the initial database search. After excluding the duplicates, 240 items entered the initial filtration by screening their titles and abstracts. Then, 227 irrelevant (not meeting the inclusion criteria) publications were directly excluded from further filtration. By reading through 13 retrieved articles, we further excluded nine articles addressing irrelevant topics and considered the remaining four studies for possible eligibility. Finally, four randomized controlled trials were included in the current systematic review (17, 18, 22, 23). It was not possible to perform a meta-analysis for the study by Tran et al. because of the lack of data or units of measurement (22). Therefore, a meta-analysis was conducted with three of included studies (17, 18, 23).

### Study characteristics

Table 1 summarized the characteristics of included studies. These studies comprised 80 patients with 16 males and 64 females. The mean age was 53.25. Each study reported WHO functional class, eight patients were in class I, 39 patients were in class II, and 23 patients were in class III in three included studies (17, 18, 22). The main types of PH were group I (pulmonary arterial hypertension, 95%) and group IV (chronic thromboembolic PH, 5%). The training protocol applied in the IMT was listed in Table 1. The training intensity performed 30% of MIP in the study of Saglam et al. and Aslan et al., Tran et al. used 30–40%, and Laoutaris et al. (23) used a high intensity (60% sustained MIP) for training. Study duration ranged from 6 to 10 weeks.

**TABLE 1:** Characteristics and training protocol of included studies.

References	Study design	Age	Male/Female	BMI	WHO FC (I/II/III/IV)	Mpap (mmHg)	Sample size	Training protocol	Outcomes
Saglam et al. (17)	RCT	49.7 ± 12.4	6/25	26.7 ± 6.1	0/16/15/0	67.3 ± 31.8	IMT: 14 CG: 15	IMT: 30% MIP; 30 min; 7 days/week; 6 weeks; CG: 10% MIP; 30 min; 7 days/week; 6 weeks;	• **Safety:** One patient suffered from muscle soreness; • **Adherence:** Good adherence • **Dyspnea and fatigue perception** ΔFSS score: IMT –6.6 ± 3.6 vs. CG 0.0 ± 3.9, *P* < 0.001**** ΔMMRC dyspnea score: IMT –0.29 ± 0.47 vs. CG 0.12 ± 0.34, *P* = 0.012 • **Respiratory muscle strength** ΔMIP: IMT 26.14 ± 12.15 vs. CG 5.87 ± 12.64, *P* < 0.001 ΔMEP: IMT 10.00 ± 8.28 vs. CG 2.80 ± 9.81, *P* = 0.004 • **Pulmonary function** FEV_1_, % predicted value: *P* = 0.013 FVC, % predicted value: *P* = 0.105 FEV_1_/FVC, %: *P* = 0.715 • **Functional capacity** 6MWT distance: *P* < 0.001 6MWT, % predicted value: *P* < 0.001 • **Quality of life** NHP-energy: *P* = 0.663 NHP-pain: *P* = 0.145 NHP-emotional reactions: *P* = 0.006 NHP-sleep: *P* = 0.707 NHP-physical mobility: *P* = 0.945 NHP-social isolation: *P* = 0.180
Laoutaris et al. (23)	RCT	54.6 ± 13.4	4/6	26.4 ± 4.7	2.6 ± 0.8	–	IMTG: 5 CG: 5	IMTG: 60% SPImax, 30 min, 3 times/week, 10 weeks;**** CG: No training	• **Maximal inspiratory pressure** IMTG 94.4 ± 16.8 vs. CG 71.4 ± 11.3 • **Inspiratory work capacity** SPImax: IMTG 412.8 ± 38 vs. CG 302 ± 61 • **Dynamic lung volumes** • **Exercise capacity** 6MWT distance: IMTG 436 ± 95 vs. CG 376 ± 109 • **Health-related quality of life** IMTG: SF36v2^®^ questionnaire, *P* = 0.004; physical functioning, *P* = 0.001; role physical, *P* = 0.002; social functioning, *P* = 0.03; reported health transition, *P* = 0.01**** CG: No significant changes
Aslan et al. (18)	RCT	48.7 ± 14.8	4/23	28.2 ± 6.4	8/12/7/0	50.1 ± 17.7	TIMT: 15 SG: 12	IMT: 30% MIP; 15 min, twice/day; 5 days/week; 8 weeks;**** SG: 9 cmH_2_O; 15 min, twice/day; 5 days/week; 8 weeks;	• **Safety:** No adverse effects • **Adherence:** Excellent adherence • **Respiratory functions** ΔMIP: TIMT 17.8 ± 4.07 vs. SG 5.41 ± 8.12, *P* = 0.023**** ΔMEP: TIMT: 6.86 ± 16.47 vs. SG –3.66 ± 13.04, *P* = 0.092 ΔFEV_1_ (%): TIMT –3.6 ± 9.7 vs. SG –4.91 ± 11.64, *P* = 0.845 ΔFVC (%): TIMT –3.46 ± 9.32 vs. SG –2.83 ± 8, *P* = 0.864 ΔFEV_1_/FVC (%): TIMT –3.53 ± 10.19 vs. SG –1.08 ± 13.26, *P* = 0.980 • **Functional exercise capacity** Δ6MWD: TIMT 23.6 ± 54.73 vs. SG 1 ± 26.24, *P* = 0.250 Δ%6MWD: TIMT 3.81 ± 10.4 vs. SG 0.53 ± 4.86, *P* = 0.282 • **Quality of life** ΔMLHFQ: TIMT –3.4 ± 6.61 vs. SG –5.91 ± 3.62, *P* = 0.240 ΔMLHFQ-physical subscore: TIMT –0.86 ± 4.3 vs. SG –2.91 ± 2.71, *P* = 0.081 ΔMLHFQ-emotional: TIMT –0.93 ± 3.82 vs. SG –1 ± 1.75, *P* = 0.883 • **Self-reported physical activity** ΔTotal energy expenditure: TIMT 446.4 ± 1772.98 vs. SG 704.91 ± 1584.61, *P* = 0.608 ΔPhysical activity duration: TIMT –12.46 ± 254.93 vs. SG 3.41 ± 63.99, *P* = 0.542 ΔActive energy expenditure: TIMT –15.66 ± 254.93 vs. SG 361.66 ± 971.39, *P* = 0.421 ΔNumber of steps: TIMT 296.86 ± 2842.56 vs. SG 700.66 ± 1411.5, *P* = 0.770
Tran et al. (22)	RCT	60 ± 14	2/10	25.1 ± 5.5	0/11/1/0	38.1 ± 10.2	IMT: 6 CG: 6	IMT: 30–40% PImax; two cycles of 30 breaths; 5 times/week; 8 weeks; CG: Continue with their routine management plan and usual daily activity	• **Compliance:** 449/460 (98%) cycles • **Average training duration:** 7.7 ± 0.8 weeks • **Pulmonary function testing** ΔFEV_1_ (% pred): –2.2 (–7.3 to 2.9), *P* = 0.35**** ΔFVC (% pred): 0.4 (–6.3 to 7.2), *P* = 0.89 ΔFEV_1_/FVC (% pred): –2.4 (–9.5 to 4.6), *P* = 0.46 • **Respiratory muscle function testing** ΔPImax: 20.5 (3.9–37.1), *P* = 0.02 ΔPImax (% pred): 23.4 (1.8–45.0), *P* = 0.04 ΔPEmax: 6.7 (–15.9 to 29.2), *P* = 0.53 ΔPEmax (% pred): 11.5 (–14.1 to 37.0), *P* = 0.34 • **Echocardiography and cardiopulmonary exercise testing** ΔPeak VO_2_: 0.4 (–2.6 to 3.4), *P* = 0.77 ΔNadir VE/VCO_2_: 0.4 (–3.8 to 4.5), *P* = 0.84 ΔOUES: 57.8 (–115.2 to 230.7), *P* = 0.47 • **Functional exercise capacity** Δ6MWD: 36.5 (3.5–69.5), *P* = 0.03 • **N-terminal pro B-type natriuretic peptide:** –9.6 (–28.8 to 9.5), *P* = 0.29

RCT, randomized control trial; BMI, body mass index; mPAP, mean pulmonary artery pressure; WHO FC, World Health Organization Functional Class; IMT, inspiratory muscle training; TIMT, threshold inspiratory muscle training; IMTG, inspiratory muscle training group; MIP, maximal inspiratory pressure; MEP, maximal expiratory pressure; PImax, maximal static inspiratory pressure; PEmax, maximal static expiratory pressure; FEV1, forced expiratory volume in 1 s; FVC, forced vital capacity; peak VO_2_, peak oxygen consumption; FSS, fatigue severity scale; MMRC, modified medical research council; 6MWT, 6 min walk test; NHP, Nottingham Health Profile; SPImax, sustained maximal inspiratory pressure; SF36v2^®^, short form health survey; CG, control group; SG, sham group; MLHFQ, Minnesota Living with Heart Failure Questionnaire; OUES, oxygen uptake efficiency slope; VE, minute ventilation; VCO2, carbon dioxide production.

### Risk of bias

**FIGURE 2:**
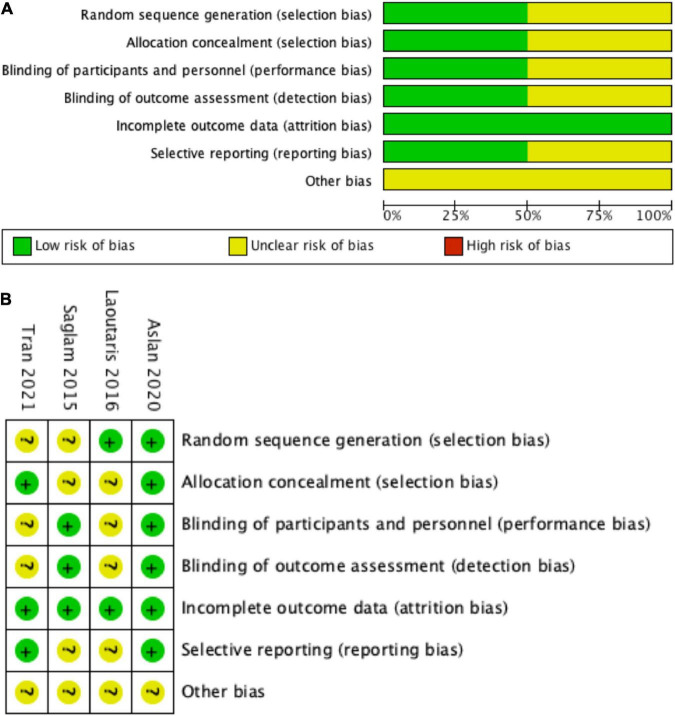
Risk of bias graph. **(A)** Review authors’ judgments about each risk of bias item presented as percentages across all included studies, **(B)** risk of bias summary: review authors’ judgments about each risk of bias item for each included study. (+) = low risk of bias; (?) = unclear; (–) = high risk of bias.

In the present systematic review and meta-analysis, the Cochrane risk of bias tool was to evaluate the quality of the included studies, and the results were presented in Figure 2. Most of the included studies described the details regarding allocation concealment, blinding of participants and personnel, blinding of outcome assessment, incomplete outcome data, and selective reporting (17, 18, 22). The other indexes of bias typically lacked specific descriptions in the included clinical studies (17, 18, 22). The study by Laoutaris et al. (23) did not report in detail the Cochrane risk of bias assessment, so the level of risk was not clear for most of the assessment components.

### Outcome measures

#### Safety and feasibility

Two of the included studies examined the safety of IMT (17, 18). Saglam et al. (17) reported one patient suffered from muscle soreness during the first treatment session, however, no patients withdrew from training during the IMT program. Saglam et al. and Aslan et al. (17, 18) found good adherence to IMT training, with patients completing the training protocol. Tran et al. (22) found compliance with the IMT protocol to be 449/460 (98%).

#### Dyspnea and fatigue perception

Only one study observed dyspnea and fatigue perception after IMT, and it was not possible to perform a meta-analysis. The results showed a significant decrease in the fatigue severity scale score in the IMT group compared with the sham group (–6.6 ± 3.6 vs. 0.0 ± 3.9, *P* = 0.012, respectively) (17). The Modified Medical Research Council dyspnea scale scores significantly improved in the IMT group compared with the sham group (–0.29 ± 0.47 vs. 0.12 ± 0.34, *P* < 0.001) (17).

#### Respiratory muscle strength

**FIGURE 3:**
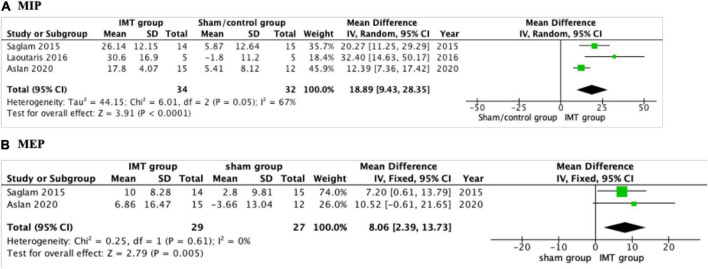
Forest plot showing the net change of maximal inspiratory pressure (MIP) and maximal expiratory pressure (MEP). **(A)** The net change of MIP, **(B)** the net change of MEP.

The effect of IMT on respiratory muscle strength was reported in all the included studies (17, 18, 22, 23). Three studies conducted a meta-analysis (17, 18, 23). MIP increased significantly following IMT with a mean net change of 18.89 cmH_2_O (95% CI: 9.43–28.35, *P* < 0.001) compared with sham/control group (Figure 3). As a low level of heterogeneity was apparent in the result (*P* = 0.05; *I^2^* = 67%), a random-effect model was applied. Tran et al. (22) found that the IMT group improved MIP by 31 cmH_2_O compared with 10 cmH_2_O in controls, *P* = 0.02. A fixed-effect model was used to analyze MEP reported by two studies (17, 18). Significant improvement was detected in MEP (8.06 cmH_2_O; 95% CI: 2.39–13.73; *P* = 0.005) compared with sham group (Figure 3).

#### Pulmonary function

**FIGURE 4:**
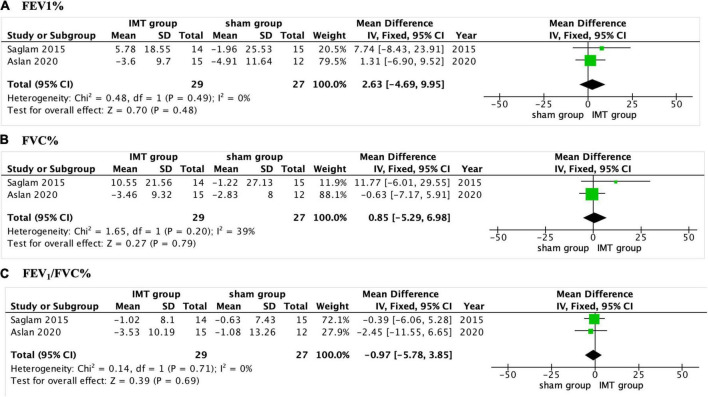
Forest plot showing the net change of pulmonary function. **(A)** The net change of FEV1%, **(B)** the net change of FVC%, and **(C)** the net change of FEV1/FVC%. FEV1, forced expiratory volume in 1 s, FVC, forced vital capacity.

Three of the included studies reported the impact of IMT on pulmonary function (17, 18, 22), and only two of them did the meta-analysis (17, 18). The pooled analysis found no significant differences in FVC%, FEV_1_%, and FEV_1_/FVC% between the IMT group and sham group (*P* > 0.05, Figure 4) (17, 18). Tran et al. (22) found there was no significant improvement in FVC%, FEV_1_%, and FEV_1_/FVC% compared with control group.

#### Exercise capacity

**FIGURE 5:**
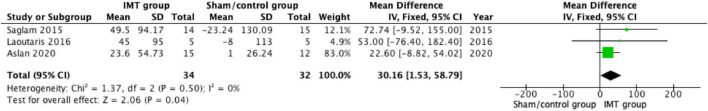
Forest plot showing the net change of 6 min walk distance (6MWD).

A 6 min walking test and cardiopulmonary exercise test were used to assess the exercise capacity in PH patients. All studies reported the impact of 6MWD (17, 18, 22, 23). Three of them did the meta-analysis and the pooled analysis found significant differences in 6MWD between the sham/control group (30.16 m; 95% CI: 1.53–58.79; *P* = 0.04) (Figure 5) (17, 18). Tran et al. (22) investigated the improvement of 6MWD (*P* < 0.001), but no significance in RestingVO_2_, PeakVO_2_, PeakHR, Oxygen pulse, VO_2_ at AT, VE/VCO_2_, oxygen uptake efficiency slope, and Peak RER compared with the control group (*P* > 0.05).

#### Quality of life

Two included studies assessed the impact of IMT on QoL (17, 18). Saglam et al. (17) used the Nottingham Health Profile (NHP) scale to assess QoL. Emotional reactions domain scores on the NHP improved significantly in the IMT group after training compared with the sham group (*P* = 0.006). No significant differences were observed between or within groups in the other domains of the NHP. Aslan et al. (18) used Minnesota Living with Heart Failure Questionnaire (MLHFQ) for the QoL assessment. The study reported that MLHFQ (*P* = 0.002) and the physical sub-scores of MLHFQ (*P* = 0.008) were improved in the sham group (18). There was a decrease in the scores of both the IMT group and the sham group after the intervention, but the comparison of the change values between the two groups was not statistically significant (*P* = 0.240). Laoutaris et al. (23) used the SF36v2^®^ questionnaire for health-related QoL assessment, found IMT significantly improved SF36v2^®^ questionnaire (*P* = 0.004) and found improved significantly physical functioning (*P* = 0.001), role physical (*P* = 0.002), social functioning (*P* = 0.03), reported health transition (*P* = 0.01) compared with control group.

## Discussion

This systematic review and meta-analysis showed that IMT is a safe, feasible, and well-tolerated physical therapy in PH patients. Furthermore, IMT has been shown to significantly improve respiratory muscle function, and exercise capacity. Only one study has reported changes in dyspnea and fatigue after training with IMT, therefore, the evidence remains insufficient. Also, the effect of this intervention on pulmonary function and QoL in PH patients remains unclear. Based on the currently limited evidence, it is reasonable to believe that IMT may be a transitional physical therapy before exercise training for PH patients. Because the improvement of respiratory muscle strength can better help PH patients adapt to the intensity of exercise training and optimize cardiac rehabilitation programs. This is also the first systematic review and meta-analysis that analyzed the effects of isolated IMT on respiratory muscle strength, exercise capacity, and QoL in PH patients (24). Finally, the impact of IMT on these outcomes remains uncertain due to the limited number of included trials, and more well-designed, large randomized controlled clinical studies are still needed.

Exercise training has been previously excluded from intervention options for PH patients due to the potential impact on the deterioration of right ventricular function as well as the potential risk of a decrease in cardiac output during intervention (25). However, this perception has been turned upside down in the last two decades as the value of exercise-based cardiac rehabilitation has been progressively demonstrated in PH patients. The ESC/ ERS presented a strong recommendation on supervised exercise training for PAH patients under optimal medical therapy (Class I, Level A) (26). As exercise training entered clinical practice, its drawbacks gradually emerged. On one hand, the acceptance of exercise training is limited in developing countries like China compared to western countries (27). Some misconceptions such as medication or surgery are the only way to address or relieve the symptoms of a disease rather than exercise. On the other hand, in the early stages of exercise training, PH patients are usually discouraged by severe dyspnea due to difficulty accepting the training intensity. From a clinical practice perspective, we consider the role of IMT may not only as an important component of physical therapy for respiratory muscle weakness but also as a transition intervention prior to formal exercise training, i.e., to optimize the dyspnea and fatigue level prior to exercise training in PH patients.

IMT has been extensively studied as a targeted approach to improving respiratory muscle weakness in cardiovascular diseases (11). Several studies have found a reduction in MIP as well as MEP in patients with PAH (13, 28). The first evidence of respiratory muscle weakness in idiopathic pulmonary arterial hypertension (IPAH) was provided in a study by Meyer et al. (28). The results showed that inspiratory and expiratory muscle weakness was independent of reduced pulmonary hemodynamics, exercise capacity, or ventilation efficiency (28). Manders et al. (29) found that chronic thromboembolic pulmonary hypertension (CTEPH) patients had reduced maximal contraction capacity of the slow-contracting muscle fibers of the diaphragm compared to controls, as well as reduced calcium sensitivity of the fast-contracting muscle fibers. These microstructural abnormalities were associated with a reduction in overall respiratory muscle strength, ultimately manifesting as a reduction in maximum “static” inspiratory pressure (29). In addition, diaphragm biopsies in PH patients show diaphragm atrophy reduced contractility, and reduced capillary density, abnormalities that may lead to defects in respiratory mechanics, i.e., the inability to change the tidal volume with increasing exercise load, resulting in increased dyspnea during exercise (30, 31). Boucly et al. (30) further demonstrated that when the inspiratory reserve is severely reduced, the inflection point in the tidal volume response marks the transition from the increased respiratory effort to unsatisfactory inspiration, a more unpleasant sensation associated with anxiety. All these results suggest that the important factor of respiratory muscle dysfunction should not be ignored in PH patients.

The results from this systematic review and meta-analysis showed that IMT significantly improved inspiratory muscle strength in PH patients in the included studies, patients with PH had an average MIP of 61.1 cmH_2_O before intervention (17, 18). According to the 2002 American Thoracic Society/ERS statement on respiratory muscle testing, a MIP of 80 cmH_2_O is required to rule out inspiratory muscle weakness (32). After 6–10 weeks of training, the MIP of PH patients in the intervention group was significantly improved. The pooled results showed a significant increase in MIP in the IMT group compared to the sham/control group (*P* < 0.001), the mean improvement was 18.89 cmH_2_O (17, 18, 23), and the average MIP values after training exceeded or reached the minimum standard of normal inspiratory muscle strength (80 cmH_2_O). This means patients with PH can achieve normal levels of inspiratory muscle function after IMT. Another interesting result is that IMT also increased expiratory muscle function in PH patients (8.06 cmH_2_O) after IMT. One possible explanation is that IMT may have also increased the patient’s expiratory capacity because the intercostal muscles, which act as expiratory muscles, have also been trained during forced ventilation (33). However, the included studies had opposite findings, so more studies are needed to further explore.

Increased dyspnea and fatigue are common occurrences in PH patients (34). This has a profound impact on participation in exercise training and QoL. Only one of our included studies reported the effect of IMT on dyspnea and fatigue in PH patients (17). The results showed that the dyspnea index and fatigue were significantly reduced in the intervention group. The possible mechanisms can be explained by the fact that IMT decreased the relative ventilatory load (increased respiratory muscle strength) (35), and/or the absolute ventilatory load (due to improvement in respiratory mechanics). However, the lack of change in spirometry in the context of improved respiratory muscle strength potentially suggests the former, more research is needed to make that conclusion. Reducing dyspnea by increasing respiratory muscle strength is a unique advantage of IMT. In addition, patients with less than 70% of the predicted MIP also showed a greater sense of dyspnea throughout exercise (36). Therefore, although the current evidence is limited, IMT may be a specialized training method to improve dyspnea and fatigue in PH patients.

Previous studies have found an association between 6MWD and long-term clinical outcomes in PH patients (37). More recently, Gabler et al. (38) found that an improvement in 6MWD > 41.8 m was associated with a lower incidence of clinical events. Therefore, improvement in 6MWD was used as a surrogate endpoint for efficacy evaluation in clinical trials in patients with PAH (39). The pooled results of our meta-analysis demonstrated the significant improvement of 6MWD after IMT when compared with the sham/control group (30.16 m, *P* = 0.04) (17, 18, 23). A possible explanation is that the improvement in inspiratory muscle strength facilitated by IMT may have helped to improve the patient’s respiratory muscle fatigue and dyspnea thereby improving the patient’s exercise capacity (40). However, our results showed improvement was limited and did not result in significant changes in all included studies. This suggests the value of IMT in physical therapy may not a substitute for exercise training, but rather plays a role in the overall intervention program as a transition prior to exercise training or as a specialized physical therapy modality to modify respiratory muscle weakness in patients with PH.

However, the limitations of our systematic review and meta-analysis should also be noted. Firstly, there are currently few clinical studies on isolated IMT in patients with PH, only four were included in this systematic review and three in the meta-analysis. However, from the limited evidence, we still found the effect of IMT on the improvement of inspiratory muscle strength and exercise capacity. Secondly, for the QoL, the evaluation methods used in each article are different, so we cannot clarify whether IMT can improve the QoL of PH patients, which is a question that needs to be further explored in follow-up studies. Thirdly, although the proportion of women over men (56–86%) (41) in patients with PAH reported in epidemiological studies appears to be consistent with the gender ratio of patients included in this systematic review and meta-analysis, we should still be aware that these results may not be representative of the value of IMT in male with PH and therefore, and this is worth exploring in future studies. Finally, as a strength training method, there is no uniform standard for the training prescription, the best mode of training and which patients benefit from this intervention are not clear. Therefore, clinical research is still needed in the future to further clarify the effect of IMT and provide new evidence for inspiratory muscle training in patients with PH.

## Conclusion

Based on the existing evidence, IMT is an effective physical therapy for increasing respiratory muscle function and exercise capacity, but still a lack of evidence on dyspnea and fatigue levels, pulmonary function, and QoL in PH patients. At the same time, we have reason to believe that IMT is a potential intervention in PH patients, which can enrich physical therapy options and serve as a bridge before formal exercise training. It is expected that IMT will play a more important role in the clinical pathway of cardiac rehabilitation for PH patients in the future. Moreover, the safety and efficacy of IMT in PH patients need to be further verified by large multi-center clinical randomized controlled trials.

## Data availability statement

The raw data supporting the conclusions of this article will be made available by the authors, without undue reservation.

## Author contributions

ZL and PY made the substantial contributions to the conception and design of the work. XZ and HQ searched, selected the materials, and extracted the data. ZL wrote the manuscript. XZ, HQ, YW, and JW revised the manuscript carefully and contributed to the statistical analysis. All authors have read and approved the final manuscript.
