# Supplementary material for: Effectiveness and safety of inspiratory muscle training in patients with pulmonary hypertension: A systematic review and meta-analysis
Source: Front Cardiovasc Med. 2022 Nov 29;9:999422. doi: 10.3389/fcvm.2022.999422 (PMC9744751; doi:10.3389/fcvm.2022.999422)
Supplement: Supplementary file 1 [file Data_Sheet_1.docx]

**Supplementary Table 1** Search strategy for PubMed.

| *#*1 | respiratory muscle training [Title/Abstract] |
| --- | --- |
| *#*2 | inspiratory muscle training [Title/Abstract] |
| *#*3 | expiratory muscle training [Title/Abstract] |
| *#*4 | breathing training [Title/Abstract] |
| *#*5 | IMT [Title/Abstract] |
| *#*6 | RMT [Title/Abstract] |
| *#*7 | *#*1 OR *#*2 OR *#*3 OR *#*4 OR *#*5 OR *#*6 |
| *#8* | pulmonary hypertension [Title/Abstract] |
| *#9* | pulmonary arterial hypertension [MeSH] |
| *#10* | pulmonary vascular disease [Title/Abstract] |
| *#11* | PH [Title/Abstract] |
| *#12* | *#8 OR #9 OR #10 OR #11* |
| *#13* | *#7* AND *#*12 |

**Supplementary Table 2** The PICOs principles of the systematic review and meta-analysis.

| P: Patients | Pulmonary hypertension |
| --- | --- |
| I: Intervention | Inspiratory muscle training |
| C: Comparison | IMT versus control/sham IMT |
| O: Outcome | / |
| S: Study | Randomized control training |
